# Pneumonia

**Published:** 1852-07

**Authors:** 


*Pneumonia.*

Pneumonia, as described by most authors, is a disease highly inflammatory in its character, requiring for its treatment venesection, purgatives, antimonials, and others of the class of remedies which, according to most writers, act favorably by moderating the so-called excessive vital action characterising this and other similar diseases.

With all due deference to the views and opinions of others upon this subject, we contend that it is unreasonable, to say the least, to regard the symptoms of disease of whatever character as indicative of too much vitality.

That the vital forces sustain life by opposing those which are merely chemical or physical, is admitted by all, hence we should naturally expect that diseased action, under whatever form, would be indicative either of diminished vital power, or of disability on the part of the organism to perform its functions harmoniously, for want of a proper balance between these powers.

Viewing the subject in this aspect, we are naturally led to inquire why depleting measures, or such as tend directly to diminish the vital forces, have been so generally recommended in pneumonia? Has success in practice, or the theoretical views of authors had the most influence in modifying practice in such cases?

That depleting treatment has not stood the test of experience is made evident by the results of numerous experiments and observations made in European hospitals, for the purpose of testing its comparative value.

Whatever may be the true pathology of pneumonia, the circumstances under which it occurs, and its symptoms, indicates, 1st, a too rapid disintegration of tissues by oxydation, and, 2d, defective action of the excretory organs, and the consequent accumulation of excrementitious matter, resulting from this disintegration, in the blood and tissues.

Such being the case, the indications in its treatment are, evidently, to prevent the too rapid destructive assimilation by diminishing the activity both of the circulation and respiration, and also to favor the elimination of the excess of excrementitious matter already accumulated.

The first of these indications is, in our opinion, best fulfilled by the use of large and repeated doses of opium or morphine; the second, by the administration of quinine, which is known to have, especially when combined with opium, a remarkable influence in exciting to action the skin, liver, kidneys, and in fact all the excreting organs.

The almost uniform success of this treatment in our practice for several years, has been such that we can now recommend it to our readers with confidence.

The following cases recently treated in the U. S. Marine Hospital with full doses of opium and quinine, will illustrate our mode of treatment and serve as examples of results:

Case I.—Thos. D., æt. 25, was admitted to the U. S. Marine Hospital, April 3d, 1852. Has severe pain in the left side—breathing short and difficult—cough troublesome, with white, glairy expectoration—auscultation reveals a subcrepitant rattle—bowels constipated.

℞.—Pulv. rhei, bi-carb. soda, aa grs. x. M. Take immediately. After it operates, give

℞.—Quin. sulph. gr. vj., morph. sulph. gr. ¼—3 times daily.

4th. Some improvement in symptoms. Passed the night with less cough and pain in the chest. Continue prescription.

8th. Unpleasant symptoms entirely removed. Discharged.

Case II.—J. B., æt. 38. Admitted April 6th. Has been sick five days. Cough severe, breathing difficult, respiratory murmur obscure over both lungs—movements of the chest restricted—coarse mucus ronchus and dullness over right side.

℞.—Quin. sulph. grs. v., morph. sulph. gr. ½. M. Give every eight hours.

7th. Not much change in physical signs. Slept better last night than had previously.

℞.—Sulph. quin. ℨ ss., pulv. opii ℈ j. M., ft. pulv. no. v., give one 3 times daily.

8th. Difficulty of breathing much relieved—cough much better. The last powder produced vomiting.

℞.—Quin. grs. vj., morph. gr. j. M. Give 3 times daily.

4 P. M. Had a severe chill—all the symptoms aggravated. Gave quin. grs. vjii., morph. gr. j.

9th, 8 A. M. Much better; bowels constipated. Give cathartic and follow after it operates, by quin. and morph., as yesterday.

10th. Respiration better; cathartic acted well. Continue quin. grs. vjii., morph. gr. ½, 3 times daily.

12th. ℞.—Pulv. rhei, bi-carb. soda, aa. grs. x., this morning, followed with quin. and. morph.

13th. Respiratory murmur free on left side, mucus rattle on the right; appetite poor; tongue furred.

℞.—Nitro-mur. acid. dilu. gtt. x., in water, 3 times daily. Continue quin. and morph. at night.

15th. Expectoration free, appetite improving.

17th. Still improving. Ordered common salt sufficient to open the bowels, and to continue acid, and quin. and morph.

20th. Improving in every respect. Still has cough; ordered cod liver oil, 1 table spoonful 3 times daily, with quin. and opii gr. j. at night. Recovery from this time rapid and complete.

Case III.—H. C., admitted April 10th. Has cough and dull, heavy pain in left side; respiration difficult; a dry, whizzing sound over left lung; tongue furred.

℞.—Quin. grs. v., opii grs. ji. M. Take 3 times daily.

11th. All the symptoms relieved. Continue treatment.

13th. Still improving. Continue and add pulv. Dov. grs. v., at bed time.

15th. Very little cough; complains of no pain; bowels constipated.

℞.—Rhei pulv., bi-carb. soda, aa. grs. x. M. Take this morning, followed by quin. grs. ji., carb. ferri grs. v., 3 times daily. He rapidly recovered. H.

